# Preterm care during the COVID-19 pandemic: A comparative risk analysis of neonatal deaths averted by kangaroo mother care versus mortality due to SARS-CoV-2 infection

**DOI:** 10.1016/j.eclinm.2021.100733

**Published:** 2021-02-15

**Authors:** Nicole Minckas, Melissa M. Medvedev, Ebunoluwa A. Adejuyigbe, Helen Brotherton, Harish Chellani, Abiy Seifu Estifanos, Chinyere Ezeaka, Abebe G. Gobezayehu, Grace Irimu, Kondwani Kawaza, Vishwajeet Kumar, Augustine Massawe, Sarmila Mazumder, Ivan Mambule, Araya Abrha Medhanyie, Elizabeth M. Molyneux, Sam Newton, Nahya Salim, Henok Tadele, Cally J. Tann, Sachiyo Yoshida, Rajiv Bahl, Suman P.N. Rao, Joy E. Lawn, COVID-19 Small and Sick Newborn Care Collaborative Group

**Affiliations:** a Department of Maternal, Newborn, Child and Adolescent Health and Ageing, World Health Organisation, Avenue Appia 20, CH-1211 Geneva 27, Switzerland; b Department of Pediatrics, University of California San Francisco, 550 16th Street, Box 1224, San Francisco, CA 94158, United States; c Maternal, Adolescent, Reproductive and Child Health Centre, London School of Hygiene and Tropical Medicine (LSHTM), Keppel Street, London WC1E 7HT, United Kingdom; d Department of Paediatrics and Child Health, Obafemi Awolowo University Teaching Hospital Complex, Ile-Ife 220005, Nigeria; e Medical Research Council Unit The Gambia at LSHTM, Atlantic Road, Fajara, The Gambia; f Department of Paediatrics, Vardhaman Mahavir Medical College and Safdarjung Hospital, Ansari Nagar, New Delhi 110029, India; g Department of Reproductive, Family and Population Health, Addis Ababa University, P.O. Box 9086, Addis Ababa, Ethiopia; h Department of Paediatrics, University of Lagos College of Medicine and Lagos University Teaching Hospital, P.M.B. 12003, Lagos, Nigeria; i Maternal and Newborn Health in Ethiopia Partnership, Emory Ethiopia, P.O. Box 793, Addis Ababa, Ethiopia; j Department of Paediatrics and Child Health, University of Nairobi and Kenyatta National Hospital, P.O. Box 19676-00202, Nairobi, Kenya; k Kenya Medical Research Institute-Wellcome Trust Research Programme, P.O. Box 43640-00100, Nairobi, Kenya; l Department of Paediatrics and Child Health, University of Malawi College of Medicine and Queen Elizabeth Central Hospital, P.B. 360, Chichiri, Blantyre 3, Malawi; m Community Empowerment Lab, 26/11 Wazir Hasan Road, Lucknow 226001, India; n Department of Paediatrics and Child Health, Muhimbili University of Health and Allied Sciences, P.O. Box 65001, Dar es Salaam, Tanzania; o Centre for Health Research and Development, Society for Applied Studies, 45 Kalu Sarai, New Delhi 110016, India; p Medical Research Council/Uganda Virus Research Institute and LSHTM Uganda Research Unit, P.O. Box 49, Entebbe, Uganda; q School of Public Health, Mekelle University, P.O. Box 1871, Mekelle, Ethiopia; r School of Public Health, Kwame Nkrumah University of Science and Technology, P.M.B. SPH-KNUST, Kumasi 00233, Ghana; s Department of Health Systems, Impact Evaluation and Policy, Ifakara Health Institute, P.O. Box 78 373, Dar es Salaam, Tanzania; t Department of Pediatrics and Child Health, Addis Ababa University, P.O. Box 9086, Addis Ababa, Ethiopia; u Department of Neonatal Medicine, University College London Hospital, 235 Euston Road, London NW1 2BU, United Kingdom; v Department of Neonatology, St John's Medical College Hospital, Sarjapur Road, Bangalore 560034, India

**Keywords:** Kangaroo mother care, Breastfeeding, Newborn, Preterm, Low birthweight, Neonatal mortality, Covid-19, SARS-CoV-2

## Abstract

**Background:**

COVID-19 is disrupting health services for mothers and newborns, particularly in low- and middle-income countries (LMIC). Preterm newborns are particularly vulnerable. We undertook analyses of the benefits of kangaroo mother care (KMC) on survival among neonates weighing ≤2000 g compared with the risk of SARS-CoV-2 acquired from infected mothers/caregivers.

**Methods:**

We modelled two scenarios over 12 months. Scenario 1 compared the survival benefits of KMC with universal coverage (99%) and mortality risk due to COVID-19. Scenario 2 estimated incremental deaths from reduced coverage and complete disruption of KMC. Projections were based on the most recent data for 127 LMICs (~90% of global births), with results aggregated into five regions.

**Findings:**

Our worst-case scenario (100% transmission) could result in 1,950 neonatal deaths from COVID-19. Conversely, 125,680 neonatal lives could be saved with universal KMC coverage. Hence, the benefit of KMC is 65-fold higher than the mortality risk of COVID-19. If recent evidence of 10% transmission was applied, the ratio would be 630-fold. We estimated a 50% reduction in KMC coverage could result in 12,570 incremental deaths and full disruption could result in 25,140 incremental deaths, representing a 2·3–4·6% increase in neonatal mortality across the 127 countries.

**Interpretation:**

The survival benefit of KMC far outweighs the small risk of death due to COVID-19. Preterm newborns are at risk, especially in LMICs where the consequences of disruptions are substantial. Policymakers and healthcare professionals need to protect services and ensure clearer messaging to keep mothers and newborns together, even if the mother is SARS-CoV-2-positive.

**Funding:**

Eunice Kennedy Shriver National Institute of Child Health & Human Development; Bill & Melinda Gates Foundation; Elma Philanthropies; Wellcome Trust; and Joint Global Health Trials scheme of Department of Health and Social Care, Department for International Development, Medical Research Council, and Wellcome Trust.

**Research in context**

**Evidence before this study**

Newborns that are preterm (15 million annually) and/or low birthweight (21 million annually) are a highly vulnerable population for whom high quality care from health services is imperative. Kangaroo mother care (KMC) involves continuous skin-to-skin contact between a newborn and a caregiver (usually the mother) and is an evidence-based intervention to improve survival among neonates weighing 2000 g or less. The COVID-19 pandemic is disrupting facility-based care. Further, mothers in facility settings are more frequently being separated from their newborns. We searched PubMed for studies published by November 12, 2020, with the search terms “COVID-19,” or “SARS-CoV-2,” or “coronavirus,” and “kangaroo mother care” [MeSH], or “care method, kangaroo mother” [MeSH], or “skin-to-skin contact” [MeSH], or “skin-to-skin care” [MeSH]. We found no peer-reviewed studies which analysed the impact of COVID-19 on KMC provision. There are conflicting global guidelines on mother-newborn care during the pandemic, particularly regarding skin-to-skin contact, and few specifically for low- and middle-income countries (LMIC). A newly published study using the British Paediatric Surveillance Unit reported 66 cases of SARS-CoV-2 among neonates receiving inpatient care in the UK between March and April 2020, of whom 17 (26%) were born to mothers with perinatal infection. Seven of these 17 neonates became infected despite being separated from their mother immediately after birth, supporting WHO, UK, and US guidance to keep mother and baby together even when maternal COVID-19 is suspected or confirmed.

**Additional value of this study**

Using the latest data from 127 LMICs and effect estimates consistent with the Lives Saved Tool, we modelled the effect of KMC on survival among neonates weighing ≤2000 g compared with the mortality risk of SARS-CoV-2 acquired through direct contact with an infected mother. An estimated 125,680 neonatal lives could be saved with universal (99%) coverage of KMC. Our most severe scenario (100% rate of SARS-CoV-2 transmission) could result in 1950 neonatal deaths from COVID-19. With a 10% rate of transmission, which is more consistent with available evidence, the number of neonatal lives lost could be as low as 200. Therefore, the benefit of providing KMC is 65 to 630 times higher than the risk of dying from COVID-19.

**Implications of all the available evidence**

Compared to the small risk of death due to COVID-19 for these vulnerable newborns, the survival advantage conferred by KMC is enormous. These data call for concerted efforts to protect facility-based newborn care and KMC during the pandemic, and to invest more in rebuilding after the pandemic, given the gaps to reach universal health coverage. Synthesising and sharing learnings regarding mitigation strategies is urgently needed to protect care for small and sick newborns and their mothers.

Alt-text: Unlabelled box

## Introduction

More than 80% of the world's 2·5 million annual neonatal deaths occur in babies with a low birthweight (LBW, <2500 g), among which two-thirds are preterm (<37 completed weeks of gestation) and one-third are small-for-gestational-age [1], [2], [3]. Prematurity complications are the leading cause of death in neonates and children aged <5 years [4]. Estimates suggest that achieving 95% coverage of special and intensive care for small and sick newborns could save nearly 750,000 neonatal lives by 2030 in 81 high-burden countries [5]. Kangaroo mother care (KMC) is part of evidence-based care for these newborns, and involves early, prolonged skin-to-skin contact and promotion of exclusive breastmilk feeding, resulting in early discharge with supportive follow-up [6]. WHO guidelines recommend KMC for all newborns weighing 2000 g or less, initiated when clinically stable [7]. The latest Cochrane review reported that neonates who received KMC had a 40% reduction in mortality, a 72% reduction in hypothermia, and a 65% reduction in severe infections compared to standard care [8].

The severe acute respiratory syndrome coronavirus-2 (SARS-CoV-2) has created a dilemma about care of mother-newborn dyads, particularly regarding KMC and breastfeeding. Evidence suggests that intrauterine mother-to-fetus transmission of SARS-CoV-2 infection is rare [9], [10], [11], [12]. Viral RNA has been detected in fetal-derived placental cells from 4 mothers with COVID-19 whose neonates tested positive on nasopharyngeal swabs at birth [9,13] and 24 h [9], 36 h [10], and 72 h [10,13] following delivery, confirming the occurrence of transplacental transmission [14]. Among 101 placentas collected in Italy during the pandemic, including 15 from COVID-19 cases, fetal tissue tested positive for SARS-CoV-2 proteins in a single placenta from a mother with COVID-19 whose newborn had early-onset pneumonia and viral positivity [10]. Three newborns had elevated levels of SARS-CoV-2 IgM antibodies shortly after birth, but repeated nasopharyngeal swabs were negative [11,15]. While nucleic acid of SARS-CoV-2 has been detected in breastmilk, there is currently no evidence of viral transmission through breastfeeding [15], [16], [17], [18]. Horizontal transmission may occur from infected family members or via nosocomial transmission in health facilities. A living systematic review reports that 4·5% of neonates born to infected mothers have tested positive for SARS-CoV-2 [19]. Across two studies including 385 neonates of SARS-CoV-2-infected mothers in the UK and New York, the overall rate of viral positivity was 3% (range: 0–5%) [12,20]. A population-wide cohort study using the British Paediatric Surveillance Unit reported 66 cases of SARS-CoV-2 among neonates receiving inpatient care in the UK between March and April 2020 (incidence 5·6 per 10,000 livebirths), of whom 17 (26%) were born to infected mothers [21]. Studies have reported mainly no symptoms or mild disease in infected newborns [11,13,21]. The risk of neonatal death due to COVID-19 appears to be very low based on existing evidence.

The COVID-19 pandemic is having substantial effects on the coverage and quality of healthcare for mothers and newborns, particularly in low- and middle-income countries (LMIC). Estimates suggest that a 45% reduction in coverage of essential interventions and decreased access to food over 6 months could result in 56,700 additional maternal deaths and 1,157,000 additional under-5 deaths [22]. This analysis did not consider disrupted care for small and sick newborns; therefore, resultant deaths could be higher. A study in Nepal during the COVID-19 lockdown reported a 52% reduction in institutional births, a 20% increase in preterm birth rates, and a three-fold increase in the facility neonatal mortality rate [24]. A meta-analysis among pregnant women with COVID-19 (30 studies, 1872 women) reported a three-fold higher risk of preterm birth (2 studies, 339 women) relative to those without the disease [23]. A rise in preterm birth rates would have a negative impact on newborns, families, and health systems worldwide, especially in LMICs. Small babies, particularly those who are preterm, are especially vulnerable to reductions in health coverage and quality.

However, as of November 12, 2020, no peer-reviewed studies which investigated the impact of the COVID-19 pandemic on KMC provision have been published. In addition, there are conflicting global guidelines on mother-newborn care during the pandemic, particularly regarding KMC and skin-to-skin contact. A systematic review of 20 clinical guidelines from 17 countries found that one-third recommend mother-newborn separation [25]. The WHO, the UK Royal College of Obstetricians and Gynaecologists, and the American Academy of Pediatrics advise that mothers who have confirmed or suspected COVID-19 should be supported to room-in with their newborns and breastfeed, if medically appropriate, while following appropriate infection control precautions [26], [27], [28]. There are very few specific guidelines for LMICs [25]. KMC and skin-to-skin contact are one of the key questions identified by Cochrane Pregnancy and Childbirth, which has no consensus among COVID-19 guidelines [19].

Hence there is an urgent need for evidence to inform KMC practice during the pandemic. In this analysis, we aimed to compare the benefits of KMC on neonatal survival during the pandemic with the risk of SARS-CoV-2 acquired through close contact with an infected mother or caregiver. We conducted a comparative risk analysis of maximum neonatal lives saved by KMC, versus maximum lives lost due to COVID-19, and incremental deaths caused by reduced KMC coverage in facilities.

## Methods

### Overview

We developed two scenarios to estimate the potential impact of the COVID-19 pandemic on facility-based preterm care, using KMC as a tracer for a neonatal special care (WHO level-2) service package [6,29]. The first scenario represents the possible benefits of practicing KMC on neonatal survival and the maximum harm that could arise from COVID-19 over 12 months. Given the limited evidence on horizontal mother-to-infant transmission of SARS-CoV-2, we conducted a sensitivity analysis to estimate additional deaths at different rates of transmission (Table 1). The second scenario estimates excess deaths reflecting real-world possibilities of a 50% coverage reduction or a full disruption of KMC in facilities. All projections were estimated for 127 LMICs, where approximately 90% of global births occur, and aggregated into five regions (appendix p 1).

**Table 1:** Inputs and risks for modelling the comparative risk of KMC versus COVID-19 for two scenarios.

	Baseline coverageˆ	Coverage reduction	SARS-CoV-2 positive mothers	Transmission rate*	Mortality risk†
1. Maximum benefit vs maximum harm	99	0	15	100/30/20/10	0•5
2. Additional deaths due to coverage reduction	20	50/100	NA‡	NA‡	NA‡

All figures are expressed as percentages.

ˆ Baseline coverage of KMC among neonates weighing ≤2000 g who are born in a facility.

⁎ Rate of SARS-CoV-2 transmission from mother or surrogate to neonate.

† Estimated mortality risk due to COVID-19 among neonates weighing ≤2000 g, based on the following assumptions: case fatality rate 0•5% [19]; KMC reduces mortality by 40% [8].

‡ NA, not applicable.

### Data inputs

National data on births and proportion of facility deliveries were extracted from the latest Lives Saved Tool (LiST) dataset (appendix p 2; https://www.livessavedtool.org). Neonatal mortality rates per country were from the WHO-Maternal and Child Epidemiology Estimation dataset (2017) [30] which is the current LiST default. National data on LBW prevalence were extracted from the WHO-UNICEF dataset (2015), including modelled estimates generated using restricted maximum likelihood and a country-level random effect for 47 countries with no input data [4]. Based on a meta-analysis of 453,000,000 births, we assumed that 34% of LBW babies are born weighing ≤2000 g (unpublished analyses, LSHTM). We applied an effect size of 40% for KMC on neonatal mortality, as reported in the latest Cochrane review [8]. To be consistent with LiST, we assumed that 58% of newborns ≤2000 g would be stable at 72 h of life and only applied the effect estimates to this affected fraction. All analyses were conducted in accordance with WHO guidelines on modelling of COVID-19 disruption impact (https://www.who.int/data/maternal-newborn-child-adolescent-aging/covid-19-data).

Evidence regarding the risk and consequences of SARS-CoV-2 infection in pregnant women and neonates is still limited. Across two studies including a total of 2458 women admitted for delivery at five hospitals in New York and one hospital in Mumbai, India, the overall rate of SARS-CoV-2 positivity was 11% (range: 8–15%) [20,31,32]. We considered that COVID-19-associated maternal and neonatal case fatality rates are 2·6% and 0·5%, respectively, according to a Cochrane living systematic review [19]. As these figures represent fatality among identified confirmed cases rather than all infected individuals, this could lead to over-estimation of deaths attributable to SARS-CoV-2.

### Full KMC coverage: Maximum benefit vs maximum harm (Scenario 1)

In scenario 1, we estimated the maximum benefit of KMC assuming 99% coverage among neonates for whom KMC is recommended as per WHO guidelines [7], meaning all neonates born in facilities weighing ≤2000 g who are stable at 72 h of life. We assumed that 60 to 80% of neonatal deaths occur in LBW neonates [3], and that 70% of these deaths are in those weighing ≤2000 g at birth [33]. We used the following formula from LiST to estimate maximum lives saved by KMC among stable neonates weighing ≤2000 g:

Mortality in neonates weighing ≤2000 g * KMC coverage

* Affected fraction (newborns stable at 72 h)

* KMC effectiveness =

LIVES SAVED DUE TO KMC

Subsequently, we estimated the number of newborns weighing ≤2000 g who would become infected and die from COVID-19 in a scenario of universal (99%) coverage of KMC. To be conservative, we applied the upper threshold of the SARS-CoV-2 positivity rate among women admitted for delivery (15%). To calculate the maximum number of lives lost due to COVID-19, we assumed a 100% rate of mother-infant transmission. We used the following equation to calculate deaths caused by COVID-19 in a full KMC coverage scenario:

Number of newborns ≤2000 g of SARS-CoV-2-positive mothers * KMC coverage

* Affected fraction

* SARS-CoV-2 transmission rate

* Infection fatality rate =

LIVES LOST DUE TO COVID-19

Transmission rates may vary in accordance with caregiver adherence to appropriate hygiene measures as well as availability and use of personal protective equipment (PPE) in facilities. Using the above equation, we conducted a sensitivity analysis considering three alternative transmission probabilities: 10% (scenario 1a), 20% (scenario 1b), and 30% (scenario 1c).

### Reduced KMC coverage: 50% reduction or full disruption (Scenario 2)

In the second scenario, we considered disruptions to KMC provision in facilities, due to fear of infection, visitation or transport restrictions, reallocation of workforce or physical space, and policy change. Coverage rates of KMC at the national level are not known. For this analysis, we projected that coverage of facility-initiated KMC is low and therefore assumed 20% global coverage of KMC before the pandemic [34]. We first considered a 50% reduction in KMC coverage from 20% to 10%. Subsequently, we assumed policy implementation that completely disrupts KMC provision, reducing coverage from 20% to 0%. Both projections were modelled for 12 months and the number of additional deaths represents the increase in deaths due to KMC coverage reductions compared with a counterfactual of no change in coverage.

Uncertainty estimates were derived at each step with a bootstrap approach by drawing 1000 samples from the input data, rerunning the model to produce estimates, taking the 2·5^th^ and 97·5^th^ percentiles of the resulting distributions as the uncertainty range, and perpetuating to the next step at the country-level. Results were grouped into five regions. This study complies with the Guidelines for Accurate and Transparent Health Estimates Reporting (GATHER) statement (appendix p 5). All analyses were conducted using Stata, version 15 (Stata Corp., College Station, Texas, USA). All data were from sources in the public domain; hence, ethical approval was not required.

### Role of the funding source

The funders played no role in study design; collection, analysis, and interpretation of data; manuscript writing; or in the decision to submit for publication.

## Results

### Full KMC coverage: Maximum benefit vs maximum harm

**Fig. 1:**
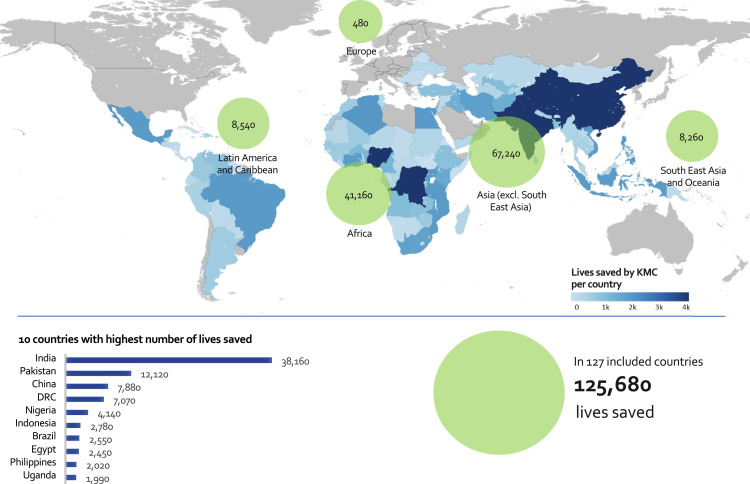
National and regional estimates of lives saved (maximum benefit) of neonates weighing ≤2000 g by KMC across 127 LMICs (100% coverage). DRC, Democratic Republic of the Congo.

**Fig. 2:**
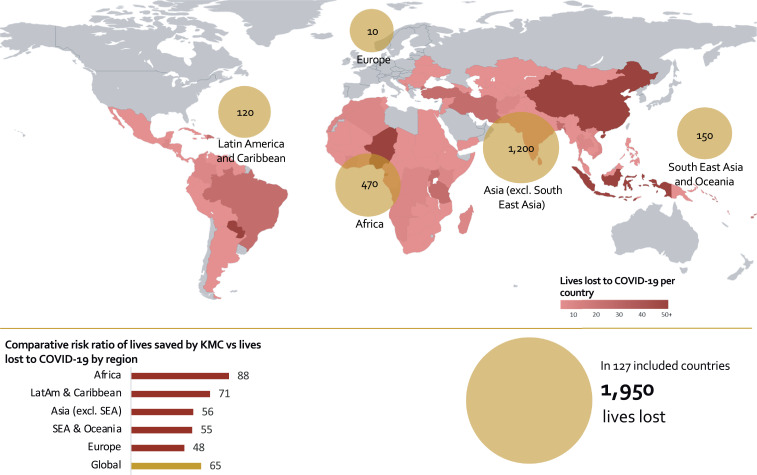
National and regional estimates of lives lost (maximum harm) among neonates weighing ≤2000 g due to COVID-19 across 127 LMICs (100% transmission). SEA, South East Asia. LatAm, Latin America. The scale of the bubbles is different than in Fig. 1.

Considering universal coverage of KMC in health facilities across the 127 countries included in this analysis, the maximum number of neonatal lives saved globally is 125,680 (lower bound: 67,710; upper bound: 243,970) over 12 months (Fig. 1). Conversely, considering a worst-case scenario of 100% mother-infant transmission of SARS-CoV-2, the maximum number of neonatal lives lost due to COVID-19 is 1950 (lower bound: 320; upper bound: 3590) over 12 months (Fig. 2). Together, Africa and Asia (excluding South East Asia) account for 108,400 (86·3%) lives saved (Fig. 1) and 1670 (85·6%) lives lost (Fig. 2).

A sensitivity analysis conducted with different rates of transmission shows that the number of lives lost due to COVID-19 could be as low as 200 (lower bound: 100; upper bound: 420), with a 10% transmission rate (Table 2). Therefore, the benefit of providing KMC is 65 to 630 times higher than the risk of dying from COVID-19 among neonates born in facilities weighing ≤2000 g. These estimates do not reflect changes in the coverage or provision of any other interventions due to pandemic-related disruptions. Moreover, COVID-19 could result in an additional 17,500 (lower bound: 3900; upper bound: 31,100) maternal deaths, which would further disrupt neonatal care and KMC in this vulnerable population.

**Table 2:** Estimated neonatal lives saved and neonatal deaths for each scenario across all modelled countries: Scenario 1 (sensitivity analysis).

	Lives saved by KMC [estimate (lb;ub)]*	Lives lost to COVID-19 [estimate (lb;ub)]*	Comparative risk ratio
1a. 10% transmission	125,680 (67,710–243,970)	200 (100–420)	630:1
1b. 20% transmission	125,680 (67,710–243,970)	390 (180–860)	NAˆ
1c. 30% transmission	125,680 (67,710–243,970)	590 (310–1230)	NAˆ
1d. 100% transmission	125,680 (67,710–243,970)	1950 (320–3590)	65:1

⁎ lb: lower bound; ub: upper bound.

ˆ NA, not applicable.

### Reduced KMC coverage: 50% reduction or full disruption

Each year, there are approximately 545,000 deaths among neonates who are born in facilities weighing ≤2000 g across the 127 countries. Our scenario of 50% coverage reduction (from 20% to 10%) would account for 12,570 (lower bound: 3960; upper bound: 21,170) additional neonatal deaths (Table 3), representing a 2·3% increase in mortality per year. These numbers could double in a situation of full disruption of KMC coverage (from 20% to 0%) following a policy change in response to the pandemic (Table 3).

**Table 3:** Additional deaths among neonates weighing ≤2000 g due to coverage reduction or full disruption of KMC in facilities, by region: Scenario 2.

	Number of countries included	Additional neonatal deaths [estimate (lb;ub)]*	
		50% reduction in KMC	Full disruption of KMC
Africa	52	4120 (2420–6880)	8230 (4430–12,310)
Europe	10	50 (20–140)	100 (24–200)
LatAm^ and Caribbean	23	850 (300–1360)	1710 (841–3380)
Asia (excluding SEA†)	25	6720 (1150–17,640)	13,450 (1640–42,420)
SEA† and Oceania	17	830 (150–1600)	1650 (490–3290)
Global	127	12,570 (3960–21,170)	25,140 (14,300–44,720)

⁎ lb: lower bound; ub: upper bound.

^ Latin America.

† South East Asia.

## Discussion

Our analyses show that even in a scenario of universal coverage of KMC, including direct breastfeeding and prolonged skin-to-skin contact, the survival benefits of KMC for neonates born in facilities weighing ≤2000 g substantially outweigh the risk of death due to COVID-19. Under our first scenario (full KMC coverage and 100% mother-infant transmission), over 12 months the maximum number of lives saved by KMC is 125,680 and the maximum number of lives lost due to COVID-19 is 1950. Africa and Asia account for 86% of estimated lives saved and lives lost, which is unsurprising given that 79% of neonatal deaths occur in these two regions [1]. Under our second scenario (KMC coverage reduction from 20% to 0%), over 12 months there would be 25,140 additional deaths, representing a 4·6% increase in neonatal mortality across the 127 countries.

Since the start of the COVID-19 pandemic and implementation of travel restrictions across the world, coverage and quality of maternal and neonatal care has deteriorated. A regional study in Italy reported a three-fold increase in stillbirths and a decrease in late preterm births (32 to <37 weeks’ gestation) during the 2020 lockdown relative to the same period in 2019 and a nationwide study in Denmark reported a reduction in extremely preterm births (<28 weeks) during lockdown compared with the previous 5 years, highlighting the indirect impact of COVID-19 on pregnancy outcomes [35,36]. There is still limited knowledge on mechanisms by which the pandemic has impacted routine care of small and sick newborns. Fear of SARS-CoV-2 transmission may result in neonates being separated from their mothers in facilities, with subsequent interruptions to skin-to-skin care and breastfeeding. Visitation restrictions might interfere with KMC, especially if mothers are unwell or relying on family members to bring food or assist with care provision. Reallocation of newborn unit staff or physical space to support the pandemic response, could lead to reductions in admission capacity and quality of care. Many LMICs adopted a centralised response strategy whereby new centres opened for COVID-19 isolation and treatment while hospitals closed their doors to routine services, severely interrupting the continuum of care for mothers and newborns. Movement restrictions, decreased transport availability, apprehension regarding COVID-19, and reluctance of caregivers to attend follow-up may reduce access to essential services. These disruptions will disproportionately affect LMIC settings, where the burden of neonatal mortality remains high. Our group recently conducted a global survey of frontline providers to explore barriers and enablers to small and sick newborn care during the pandemic. The findings suggested that evidence-based practices are being disrupted, with two-thirds of respondents reporting they do not allow mothers with confirmed or suspected COVID-19 to practice routine KMC and nearly one-quarter reporting they do not allow breastmilk feeding, even by uninfected caregivers [38]. Further, 20% reported changes in KMC practice among SARS-CoV-2-negative mothers and 7% reported complete disruption of KMC services. Respondents highlighted the importance of caregiver counselling on hygiene precautions, ensuring provision of adequate PPE, and promoting clear guidance regarding KMC and breastfeeding [37].

Although it may be difficult to completely mitigate the risk of SARS-CoV-2 transmission considering the close proximity of the mother and baby during skin-to-skin contact, infection prevention and control practices have been shown to reduce that risk considerably. A study among 120 newborns of infected mothers in New York reported that none tested positive for SARS-CoV-2 through 14 days when kept in a closed isolette in the mother's room and held for feeding [20], suggesting that rooming-in and breastfeeding are safe when accompanied by mask-wearing and hygiene precautions [20]. Guidance from Newborn Essential Solutions and Technologies for hospital-based newborn care in LMICs promotes that babies of mothers with suspected or confirmed COVID-19 should receive skin-to-skin contact and breastmilk feeding, with mothers practicing frequent hand and respiratory hygiene, including wearing a mask when near the baby https://www.nest360.org/covid-19-resources [45]. Further, a population-level study in the UK reported that neonatal infection in the first 7 days following birth to a SARS-CoV-2-positive mother was uncommon and generally mild, despite national guidance that promoted keeping mothers and babies together and breastfeeding, with appropriate hygiene precautions [21]. Prolonged hospital stays may increase the risk of nosocomial infection for both the baby and the mother. Adequate PPE, effective testing of staff and families, and strict adherence to infection control measures could mitigate these risks to some extent. In addition, the potential implications of accelerating discharge beyond the routine early discharge criteria for KMC should be considered.

Postnatal transmission of SARS-CoV-2 could also occur through breastfeeding; however, this has yet to be confirmed. Breastfeeding transmission would require exposure to infectious breastmilk as well as acquisition via an oral or gastrointestinal route. SARS-CoV-2 RNA has been detected in breastmilk in rare cases [7/141 samples (5%) in 64 women] [15], [16], [17], [18]. One case series reported a negative viral culture of a single RNA-positive milk sample, suggesting that SARS-CoV-2 does not represent replication-competent virus capable of infecting the baby [16]. Studies have reported two infected neonates who received SARS-CoV-2-positive breastmilk [17,18]. Both tested positive after the onset of maternal symptoms whilst rooming-in together; thus, horizontal transmission could not be excluded [17,18]. Further, one neonate had two subsequent negative tests despite continuing to exclusively breastfeed whilst the mother was positive [17], supporting the view that breastmilk is not a source of infection. Breastmilk has immunological and antimicrobial components that protect against many invasive pathogens; however, its mitigatory effects against SARS-CoV-2 have yet to be determined. Moreover, the long-term benefits of breastfeeding on health, growth, and cognitive development must also be taken into account when determining public health policies regarding KMC, mother-newborn separation, and infant feeding.

A strength of this analysis is that it is the first to quantify the potential impact on neonatal survival of KMC compared to coverage disruptions due to COVID-19. We use transparent outlines and conservative assumptions, and perpetuate uncertainty throughout our modelling, giving realistically wider uncertainty bounds. Our scenarios should be understood as estimates to help guide practitioners and policymakers, rather than precise predictions of the effects of KMC disruption on neonatal survival. Given the lack of reliable country-level data, we assumed universal coverage of KMC to compare the survival benefit relative to the risk of death due to COVID-19. Our KMC coverage assumptions do not consider the timeliness, components, or quality of service provision in accordance with WHO guidelines [7], nor do they account for caregiver adherence to provider instructions, e.g., duration of skin-to-skin contact [29]. We do not present country-specific estimates because LBW prevalence is not publicly available for 47 countries in the WHO-UNICEF dataset [3]. Our estimates are conservative since we have used the worst-case COVID-19 mortality impact yet the least effects for KMC, only considering survival benefit, not disability-adjusted life years or developmental outcomes. Furthermore, we did not consider the additional impact of initiating KMC before stabilisation [38], [39], [40] or implementation at a community-level [41]. Hence these estimates might underestimate the potential magnitude of these benefits and also other evidence-based newborn care practices, such as delayed cord clamping [42]. Additional studies are underway to provide more precise evidence on SARS-CoV-2 transmission from mother to newborn [43].

While COVID-19 is widely recognised to be reversing some of the hard-won health gains over recent decades, including for neonates, our findings highlight the importance of protecting and promoting KMC and routine health services for mothers and newborns during and in the aftermath of the pandemic. Despite clear evidence of impact in improving survival among LBW newborns [8], KMC scale-up has been slow, hindered by lack of national investments to implement at scale, and hard to track given lack of coverage data in routine health information systems [34]. Investments in maternal, neonatal, and early childhood interventions have lifelong and intergenerational health, developmental, and economic benefits [44].

KMC is a life-saving intervention for neonates, as well as a cornerstone of family-centred care. Compared to the small mortality risk due to COVID-19, the survival benefit conferred by KMC is enormous. KMC and breastfeeding should be encouraged for all mothers and newborns, including among mothers with confirmed or suspected COVID-19. If mothers are unwell, healthy family members may provide KMC. Synthesis and sharing of learnings are urgently needed regarding resilience and innovations to protect newborns in the pandemic and beyond. Preterm and LBW neonates are our most vulnerable citizens and ensuring provision of essential services should be a priority. The post-pandemic recovery period presents opportunities to rebuild and invest in achieving universal coverage of high-quality maternal and child health services, with a focus on small and sick newborn care including KMC.

## Declaration of Interests

We declare no competing interests.

## Appendix Supplementary materials

Image, application 1
