# Supplementary material for: Preterm care during the COVID-19 pandemic: A comparative risk analysis of neonatal deaths averted by kangaroo mother care versus mortality due to SARS-CoV-2 infection
Source: eClinicalMedicine. 2021 Feb 15;33:100733. doi: 10.1016/j.eclinm.2021.100733 (PMC7955179; doi:10.1016/j.eclinm.2021.100733)
Supplement: Supplementary file 1 [file mmc1.pdf]

Appendix to "Preterm care during the COVID-19 pandemic: a comparative risk analysis of neonatal deaths averted by kangaroo mother care versus mortality due to SARS-CoV-2 infection"

Table S1. Grouping of countries by region

Africa		Europe	Latin America and Caribbean
Algeria	Democratic Republic of Congo	Albania	Belize
Egypt	Equatorial Guinea	Belarus	Costa Rica
Morocco	Gabon	Bosnia and Herzegovina	El Salvador
Sudan	São Tomé and Príncipe	Bulgaria	Guatemala
Tunisia	Burundi	Montenegro	Honduras
Benin	Comoros	Moldova (Republic of)	Mexico
Burkina Faso	Djibouti	North Macedonia[*]	Nicaragua
Cabo Verde	Eritrea	Romania	Panama
Côte d'Ivoire	Ethiopia	Serbia	Haiti
Gambia	Kenya	Ukraine	Cuba
Ghana	Madagascar		Dominican Republic
Guinea	Malawi		Jamaica
Guinea-Bissau	Mauritius		Saint Lucia
Liberia	Mozambique		Guyana
Mali	Rwanda		Suriname
Mauritania	Somalia		Argentina
Niger	South Sudan		Bolivia
Nigeria	Uganda		Brazil
Senegal	United Republic of Tanzania		Colombia
Sierra Leone	Zambia		Ecuador
Togo	Zimbabwe		Paraguay
Angola	Botswana		Peru
Cameroon	Lesotho		Venezuela
Central African Republic	Namibia		
Chad	South Africa		
Congo	Swaziland		
**Asia (excluding Southeast Asia)**		**Oceania and Southeast Asia**	
Armenia	Yemen	Cambodia	Solomon Islands
Azerbaijan	Afghanistan	Indonesia	Timor-Leste
Georgia	Bangladesh	Lao[†]	Tonga
Jordan	Bhutan	Malaysia	Vanuatu
Iran (Islamic Republic of)	India	Myanmar	
Iraq	Maldives	Philippines	
Kazakhstan	Nepal	Thailand	
Kyrgyzstan	Pakistan	Viet Nam	
Syrian Arab Republic	Sri Lanka	Fiji	
Tajikistan	China	Kiribati	
Turkey	Korea[^]	Micronesia[‡]	
Turkmenistan	Mongolia	Papua New Guinea	
Uzbekistan		Samoa	

[*]Republic of North Macedonia. [^]Democratic People's Republic of Korea. [†]Lao People's Democratic Republic. [‡]Federated States of Micronesia.

Table S2. National data sources

Country	Facility birth proportion	Low birthweight prevalence
Afghanistan	DHS 2015	No input data
Albania	DHS 2017-2018	DHS 2008; MICS 2000, 2005; National Statistical Office 2000-2010; General Directory of Civil Status 2012-2015
Algeria	MICS 2012	MICS 2012
Angola	DHS 2015	MICS 2001
Argentina	MICS 2011	National Statistical Office 2000-2015
Armenia	DHS 2015-2016	DHS 2000, 2005, 2010, 2015; Ministry of Health 2000-2015
Azerbaijan	DHS 2016	DHS 2006; National Statistical Office 2000-2013
Bangladesh	MICS 2019	MICS 2013
Belarus	MICS 2012	MICS 2005, 2012; National Statistical Office 2000-2014
Belize	MICS 2015	MICS 2006
Benin	DHS 2017-2018	DHS 2001, 2006, 2011, 2014
Bhutan	MICS 2010	MICS 2010
Bolivia	DHS 2008	DHS 1998, 2003, 2008; MICS 2000
Bosnia and Herzegovina	MICS 2011	MICS 2006, 2011
Botswana	Family Health Survey 2007	National Statistical Office 2000-2010
Brazil	PHDS 2006	Ministry of Health 2000-2014; SINASC 2015
Bulgaria	No household data	National Statistical Office 2000-2015
Burkina Faso	DHS 2010	MICS 2006; DHS 2010; National Statistical Office 2011-2015
Burundi	DHS 2016	MICS 2000; DHS 2010
Cabo Verde	DHS 2005	No input data
Cambodia	DHS 2014	DHS 2005, 2010, 2014
Cameroon	MICS 2014	DHS 1998, 2004; MICS 2000, 2006, 2014
Central African Republic	MICS 2010	MICS 2000, 2006, 2010
Chad	DHS 2014	No input data
China	No household data	Survey 2008, 2013
Colombia	DHS 2015	DHS 2000, 2005, 2010, 2015; National Statistical Office 2000-2015
Comoros	DHS 2012	MICS 2000; DHS 2012
Congo	MICS 2014	DHS 2005, 2011
Costa Rica	MICS 2011	MICS 2011; National Statistical Office 2001, 2003-2015
Cuba	MICS 2014	MICS 2014; National Statistical Office 2000-2015
Côte d'Ivoire	MICS 2016	DHS 1998, 2011; MICS 2000, 2006
Democratic People's Republic of Korea	MICS 2017	No input data
Democratic Republic of the Congo	DHS 2013	MICS 2001, 2010; DHS 2007, 2013
Djibouti	MICS 2006	No input data
Dominican Republic	MICS 2014	DHS 1999, 2002, 2007, 2013; MICS 2000, 2014
Ecuador	NSS 2012	National Statistical Office 2009-2015
Egypt	DHS 2014	No input data
El Salvador	MICS 2014	MICS 2014; National Statistical Office 2009-2015
Equatorial Guinea	DHS 2011	No input data
Eritrea	DHS 2002	No input data
Ethiopia	DHS 2016	No input data
Fiji	No household data	No input data
Gabon	DHS 2012	DHS 2000, 2012
Gambia	MICS 2018	DHS 2013

Country	Facility birth proportion	Low birthweight prevalence
Georgia	MICS 2018	MICS 2005; National Statistical Office 2000-2015
Ghana	DHS 2014	MICS 2006, 2011; DHS 2008, 2014
Guatemala	DHS 2014	DHS 1999, 2015; National Statistical Office 2009-2015
Guinea	DHS 2018	No input data
Guinea-Bissau	MICS 2014	MICS 2006, 2014
Guyana	MICS 2014	MICS 2000, 2006, 2014; DHS 2009
Haiti	DHS 2016	No input data
Honduras	DHS 2011	DHS 2005, 2011
India	DHS 2015	National Family Health Survey 2005-2006, 2015-2016†
Indonesia	DHS 2012	DHS 1997, 2002, 2007, 2012
Iran (Islamic Republic of)	No household data	No input data
Iraq	MICS 2018	No input data
Jamaica	MICS 2011	MICS 2005, 2011; Ministry of Health 2005
Jordan	PFHS 2017-2018	DHS 1997, 2002, 2007, 2009, 2012
Kazakhstan	MICS 2015	DHS 1999; MICS 2006, 2010, 2015; National Statistical Office 2000-2014
Kenya	DHS 2014	DHS 1998, 2003, 2014
Kiribati	DHS 2009	No input data
Kyrgyzstan	MICS 2018	DHS 1997, 2012; MICS 2005, 2014; Ministry of Health 2004-2015
Lao People's Democratic Republic	MICS 2017	MICS 2011
Lesotho	MICS 2018	MICS 2000; DHS 2004, 2009, 2014
Liberia	DHS 2013	No input data
Madagascar	MDG Survey 2012	DHS 1997, 2003, 2008; MICS 2000
Malawi	DHS 2015	DHS 2000, 2004, 2010, 2016; MICS 2006, 2013
Malaysia	No household data	Consultation 2001-2015
Maldives	DHS 2016-2017	Ministry of Health 2007-2012; administrative data 2014
Mali	MICS 2015	No input data
Mauritania	MICS 2015	No input data
Mauritius	No household data	National Statistical Office 2000-2015
Mexico	MICS 2015	MICS 2015; National Statistical Office 2008-2015
Micronesia (Federated States of)	No household data	No input data
Mongolia	MICS 2018	MICS 2000, 2005, 2010, 2014
Montenegro	MICS 2018	MICS 2005, 2013; Institute of Public Health 2000-2015
Morocco	DHS 2003	DHS 2003
Mozambique	DHS 2015	DHS 1997, 2003, 2011
Myanmar	DHS 2015	DHS 2015
Namibia	DHS 2013	DHS 2000, 2006, 2013
Nepal	DHS 2016	DHS 2011, 2016; MICS 2014
Nicaragua	RHS 2006	DHS 1998, 2001
Niger	DHS 2012	No input data
Nigeria	DHS 2018	No input data
Pakistan	DHS 2017-2018	No input data
Panama	MICS 2013	MICS 2013; National Statistical Office 2000-2004, 2006-2015
Papua New Guinea	DHS 2016-2018	No input data
Paraguay	MICS 2016	MICS 2016; National Statistical Office 2014-2015
Peru	DHS 2016	DHS 2000, 2004, 2008-2012

Country	Facility birth proportion	Low birthweight prevalence
Philippines	DHS 2017	DHS 1998, 2003, 2008, 2013
Republic of Moldova	MICS 2012	DHS 2005; MICS 2012; National Statistical Office 2002-2015
Republic of North Macedonia	MICS 2011	MICS 2005, 2011; National Statistical Office 2000-2015
Romania	No household data	National Statistical Office 2000-2015
Rwanda	DHS 2014	DHS 2005, 2010, 2014
Saint Lucia	MICS 2012	No input data
Samoa	DHS 2009	No input data
Senegal	DHS 2017	DHS 2005, 2010, 2012, 2014-2016
Serbia	MICS 2014	MICS 2005, 2010, 2014
Sierra Leone	MICS 2017	DHS 2008, 2013; MICS 2010
Solomon Islands	DHS 2006	No input data
Somalia	MICS 2006	No input data
South Africa	DHS 2016	DHS 1998; HMIS 2005-2012, 2014
South Sudan	MICS 2010	No input data
Sri Lanka	DHS 2016	Routine hospital-based information system 2001-2003, 2005-2015
Sudan	MICS 2014	No input data
Suriname	MICS 2010	MICS 2000, 2006, 2010; National Statistical Office 2007-2008, 2011-2014
Swaziland	MICS 2014	MICS 2000, 2010, 2014; DHS 2006
Syrian Arab Republic	MICS 2006	No input data
São Tomé and Príncipe	MICS 2014	National Statistical Office 2012-2015
Tajikistan	DHS 2017-2018	MICS 2000, 2005; DHS 2012; National Statistical Office 2000-2014
Thailand	MICS 2015	MICS 2005, 2012; National Statistical Office 2001-2010; Ministry of Interior 2011; Health Information Unit 2012-2015
Timor-Leste	DHS 2016	No input data
Togo	DHS 2013	MICS 2000, 2006; DHS 2013
Tonga	DHS 2012	No input data
Tunisia	MICS 2018	MICS 2011
Turkey	DHS 2013	DHS 1998, 2003; Ministry of Health 2012, 2014-2015; National Statistical Office 2013
Turkmenistan	MICS 2015	MICS 2015; National Statistical Office 2002-2010; Ministry of Health 2011-2015
Uganda	DHS 2016	No input data
Ukraine	MICS 2012	MICS 2005, 2012; DHS 2007; National Statistical Office 2000-2013
United Republic of Tanzania	DHS 2015	DHS 1999, 2004, 2010, 2015
Uzbekistan	MICS 2006	MICS 2000, 2006; National Statistical Office 2000-2014
Vanuatu	DHS 2013	MICS 2007
Venezuela	No household data	National Statistical Office 2000-2015
Viet Nam	MICS 2013-2014	DHS 1997, 2002; MICS 2000, 2010, 2013
Yemen	DHS 2013	No input data
Zambia	DHS 2018	MICS 1999; DHS 2001, 2007, 2013
Zimbabwe	MICS 2019	DHS 1999, 2005, 2010, 2015; MICS 2006, 2014

[†]Only partial data were available for the most recent National Family Health Survey in India.

Table S3. GATHER checklist of information that should be included in reports of global health estimates, with description of compliance and location of information for "Preterm care during the COVID-19 pandemic: a comparative risk analysis of neonatal deaths averted by kangaroo mother care versus mortality due to SARS-CoV-2 infection"

#	GATHER checklist item	Description of compliance	Reference
**Objectives and funding**			
**1**	Define the indicator(s), populations (including age, sex, and geographic entities), and time period(s) for which estimates were made.	Narrative provided in paper describing indicators, definitions, populations, and time periods	Main text (Methods)
**2**	List the funding sources for the work.	Funding sources listed in paper	Summary (Funding)
**Data Inputs**			
For all data inputs from multiple sources that are synthesized as part of the study:			
**3**	Describe how the data were identified and how the data were accessed.	Narrative description of data seeking methods provided in paper	Main text (Methods)
**4**	Specify the inclusion and exclusion criteria. Identify all ad-hoc exclusions.	Narrative about inclusion and exclusion criteria by data type provided in paper	Main text (Methods)
**5**	Provide information on all included data sources and their main characteristics. For each data source used, report reference information or contact name/institution, population represented, data collection method, year(s) of data collection, sex and age range, diagnostic criteria or measurement method, and sample size, as relevant.	Narrative description and list of all data sources provided in submission materials	Main text (Methods), Appendix (pg 2-4), online data sources
**6**	Identify and describe any categories of input data that have potentially important biases (e.g., based on characteristics listed in item 5).	Summary of known biases included in paper	Main text (Methods)
For data inputs that contribute to the analysis but were not synthesized as part of the study:			
**7**	Describe and give sources for any other data inputs.	Narrative description and sources for other data inputs provided in submission materials	Main text (Methods), online data sources
For all data inputs:			
**8**	Provide all data inputs in a file format from which data can be efficiently extracted (e.g., a spreadsheet rather than a PDF), including all relevant meta-data listed in item 5. For any data inputs that cannot be shared because of ethical or legal reasons, such as third-party ownership, provide a contact name or the name of the institution that retains the right to the data.	Downloads of input data are available through online data sources	Online data sources: https://www.livessavedtool.org; https://www.who.int/healthinfo/global_burden_disease/estimates/en/index2.html; https://data.unicef.org/resources/unicef-who-low-birthweight-estimates-levels-and-trends-2000-2015; https://cgf.cochrane.org/news/covid-19-coronavirus-disease-fertility-and-pregnancy
**Data analysis**			
**9**	Provide a conceptual overview of the data analysis method. A diagram may be helpful.	Overview of overall methodological process and description of the process is provided	Main text (Methods)
**10**	Provide a detailed description of all steps of the analysis, including mathematical formulae. This description should cover, as relevant, data cleaning, data pre-processing, data adjustments and weighting of data sources, and mathematical or statistical model(s).	Detailed description of all steps of analysis, including mathematical formulae, is provided in the paper	Main text (Methods)
**11**	Describe how candidate models were evaluated and how the final model(s) were selected.	Provided in the paper	Main text (Methods)

**12**	Provide the results of an evaluation of model performance, if done, as well as the results of any relevant sensitivity analysis.	Provided in the paper	Main text (Results)
**13**	Describe methods for calculating uncertainty of the estimates. State which sources of uncertainty were, and were not, accounted for in the uncertainty analysis.	Provided in the paper	Main text (Methods)
**14**	State how analytic or statistical source code used to generate estimates can be accessed.	Access statement provided	Statistical source code used to generate estimates will be made available upon request
**Results and Discussion**			
**15**	Provide published estimates in a file format from which data can be efficiently extracted.	Included in the paper; electronic file is not provided given only a few reported estimates (5 world regions and a single time period)	Main text (Results)
**16**	Report a quantitative measure of the uncertainty of the estimates (e.g., uncertainty intervals).	Uncertainty intervals are provided with all results	Main text (Results)
**17**	Interpret results in light of existing evidence. If updating a previous set of estimates, describe the reasons for changes in estimates.	Interpretation of results in light of existing evidence is provided in the narrative of paper	Main text (Research in Context and Discussion)
**18**	Discuss limitations of the estimates. Include a discussion of any modelling assumptions or data limitations that affect interpretation of the estimates.	Discussion of limitations is provided in the narrative of paper	Main text (Discussion)
